# Topic: Arthropod Biodiversity: Ecological and Functional Aspects

**DOI:** 10.3390/insects15100766

**Published:** 2024-10-04

**Authors:** Giorgia Sollai, Anita Giglio, Piero G. Giulianini, Roberto Crnjar, Paolo Solari

**Affiliations:** 1 Department of Biomedical Sciences, University of Cagliari, 09042 Monserrato, Italy; crnjar@unica.it; 2 Department of Biology, Ecology and Earth Science, University of Calabria, 87036 Rende, Italy; anita.giglio@unical.it; 3 Department of Life Sciences, University of Trieste, 34127 Trieste, Italy; giulianini@units.it

Invertebrate animals with a segmented body, exoskeleton, and articulated appendages represent the largest phylum in the animal kingdom, Arthropoda, and account for over 80% of all known living species. They exhibit great biodiversity with a wide range of adaptations and forms such as insects, lobsters, crabs, spiders, scorpions, mites, centipedes, and millipedes that live in every habitat on earth. Arthropods play an extremely important role in maintaining ecosystem services, including benefits to humans [1,2]. For example, many species pollinate plants, produce useful substances, serve as pest control, and serve as food for other animals in most trophic webs [3,4,5]. Moreover, mites, isopods, myriapods, and insects are scavengers or decomposers that break down dead plants and animal material, converting them into soil nutrients [6], or are valued bioindicators of environmental pollution [7,8,9]. Many crustacean species (crabs, lobsters, shrimps, and crayfish) are largely consumed by humans and are therefore farmed on an intensive commercial scale [10]. In contrast, other crustaceans and insects are highly invasive species and represent one of the greatest threats to biodiversity worldwide, requiring strict control strategies [11,12,13,14,15,16]. Others are direct pests of crops and stored products [17], hematophagous vectors, or intermediate hosts of pathogenic organisms [18].

This interdisciplinary topic provided a platform to highlight new research findings and significant advances in morphological and functional adaptations and ecology, diversity, and conservation of arthropods. We reviewed 48 articles published in peer-reviewed journals, including 29 articles (27 original and 2 reviews) published in *Insects*, 11 in *Diversity* (10 original articles and 1 review), 5 in *Animals,* and 3 articles in *Life*.

The range of species, whether important indicators of ecosystem health, invasive species, or disease vectors, depends strongly on their ability to adapt to environmental and climatic conditions, as well as on the availability of suitable hosts in both natural and anthropized environments. In this regard, the interaction of species with the environment in which they live, be it natural or anthropogenic, morphofunctional adaptations, and genetic traits are the common thread of the 29 papers published in Insects. Olszewski et al. [19], aiming to determine the species composition of digger wasp communities (Spheciformes) in dispersed psammophilous grasslands of river valley environments in northern Poland, confirmed the findings of other research that the number of digger wasp species decreases with increasing woodland cover [20]. This study suggested that, from the point of view of biodiversity protection, the management of sites of significant value should, above all, preserve the mosaicity of habitats. The aim of the study by Munguia-Soto et al. [21] was to compare the population abundance and density of wild bee species over a four-year period to assess potential trends, threats, and factors favouring bee populations in the southern Chihuahuan Desert, highlighting the importance of pan trap colour, year, season, and species to evaluate bee abundance. In another study aimed at filling a gap in information on river ecosystems and their associated aquatic fauna, Gómez-Marín et al. [22] investigated the freshwater macroinvertebrate communities of Los Tuxtlas, Veracruz, Mexico. These authors reported a high diversity, with organisms belonging to seven phyla, nine (sub)classes, 21 (sub)orders and 65 families, providing elements for their conservation, management, and restoration.

On the other hand, the transformation of forests into agricultural and livestock systems can negatively affect the ecological dynamics and ecosystem services provided by different groups of insects. In this respect, the study by Tovar et al. [23] in the Caribbean region of Colombia showed that using Ivermectin in the antiparasitic management of cattle generates short- and long-term effects on the richness, abundance, biomass, and functional groups of dung beetles in livestock systems. This can slow down the recycling processes of cattle manure and lead to an accumulation of dung on pastures, thus affecting soil fertility, structure, and aeration, as well as plant species diversity in paddocks, due to non-secondary seed dispersal [24] and less control of biting flies and parasites affecting livestock [25]. Therefore, these authors suggest using integrated treatment management to prevent the recycling fauna from being affected.

An innovative technique for studying the factors that determine the structure of populations has been proposed by García-Meseguer and colleagues applying bioinformatic tools to assemble genomic data and detect the mitochondrial genome in order to delve deeper into various aspects, such as identifying cryptic species, exploring their genetic diversity, investigating potential sex-biassed dispersal, and assessing the genetic structure and connectivity of their populations [26].

Since automatic video tracking of the activity/movement of an experimental organism is essential for reliable and repeatable quantitative analyses in behavioural ecology, Málik-Roffa et al. [27] have proposed an easy-to-use open-source software called *BugTracker*, which is compatible with Windows, Linux, and MacOS operating systems, and can reliably track studied organisms of any size and speed. It analyses videos under highly variable lighting conditions using a more robust and complex tracking algorithm than threshold- or contrast-based alternatives [28,29,30].

Factors that can influence population size and distribution of populations include altitude, climate, and associated ecological differences, such as the availability of host plants. Flinte et al. [31] observed that the species richness of the Darwin wasp subfamily Pimplinae along an altitudinal gradient on a mountain in a tropical biodiversity hotspot such as the Brazilian Atlantic Rainforest is consistent with the existence of a typical latitudinal gradient. They provided strong evidence that alpha diversity and richness are lowest at high elevations and peak at low to intermediate elevations at least in one location, but that high species turnover can occur at all elevations. In deciduous forests of central European Russia, Gornostaev et al. [32] studied the vertical distribution of fruit fly species having different ecological preferences and found that species diversity varied between different sites and levels during the season. They described five different types of seasonal vertical distributions, showing that drosophilid mycetobiont species prefer the lower level of the forest, while xylosaprobionts prefer the tree canopy. A significant influence of the “site” factor was detected only for three species, while more than half of the species showed dependence of abundance on season and level, and almost all species showed it through the interaction of these factors. Maximum species diversity was observed at the beginning of June and September, while a significant decrease in diversity occurred at the end of July and the end of August.

Natural grasslands provide a wide range of ecosystem services, including provisioning, regulating, cultural, and supporting services. However, intense anthropic activities such as grazing can affect soil mesofauna, including Acari, Collembola, and Nematoda involved in the decomposition of soil organic matter, nutrient cycling, and soil structure. Indeed, the lowest species richness of soil mite communities was found in intensively grazed grasslands compared to ungrazed ecosystems in the Făgăraş Mountains, Romania [33] due to the influence of environmental variables including air temperature and humidity, soil temperature and moisture content, and vegetation cover. These factors affect resistance to soil penetration, which is significantly higher in intensively grazed grasslands, reducing the structure of the mite community. Li et al. [34] studied the vertical distribution of the springtail community and its environmental liability in the coastal mudflat wetlands of the Yangtze River Delta (China). They found that seasonal fluctuations in the vertical distribution of the collembolan species *Spartina alterniflora*, especially in summer, suggest that the developed furcula may also support the survival of springtail species. Song et al. [35] investigated the influence of climate and grazing on insect diversity in two types of grasslands along the Eastern Eurasian Steppe Transect (EEST) and examined whether the effects were mediated by changes in plant diversity. They found that both climate and grazing affected insect communities by mediating plant diversity, supporting the importance of bottom-up effects in these ecosystems. The relative contributions of specific plant traits varied by steppe type and insect functional group, with typical steppes and herbivores responding more strongly to vegetation diversity.

Climate changes are known to influence the growth conditions and distribution range of tree species in Europe [36], which may affect the communities of different organisms associated with these tree species. *Quercus variabilis* was used as a model to study the relationships between altitude and the species composition and diversity of leafminers [37]. The study showed that different leaf-mining insect groups could adapt to different altitudes, and the hump-shaped distribution pattern was typical of leaf-miner diversity along the altitude gradient. In the study on *Aceria angustifoliae* [38], parameters such as population fluctuation in buds and on leaves, emergence and migration to overwintering sites, and temperature-dependent emergence from overwintering sites were evaluated. Population density on leaves was found to increase in summer and reach a higher and later peak in 2018, then gradually decreased in autumn as the mites migrated to the overwintering sites.

The study by Lynikienė et al. [39] investigated whether *Larix* sp. could provide suitable habitats for insects and lichens associated with *Picea abies* to maintain their biodiversity under climate change. This study revealed that *P. abies* and *Larix* sp. share a large number of phylogenetically related insect and lichen species. As climate change is expected to have a strong negative impact on *P. abies* in the forest ecosystems of Eurasia, its gradual replacement by coniferous trees such as *Larix* sp. is suitable to provide alternative habitats for insects and lichens, thus supporting forest biodiversity. Anttonen et al. [40] studied how the nutrient content of tree leaves affects the trait composition of caterpillar communities and how this relationship is modified by the abundance of surrounding trees and seasonality. They found that the effects of tree richness, season, and leaf characteristics predictably influence the species diversity and trait composition of immature herbivorous insects in naturally assembled herbivore communities. The influence of plant richness on herbivore traits was shown to affect the species pool already at fine spatial scales and differs from effects observed at larger scales. The survival of the offspring is closely linked to the correct choice of host by egg-laying females [41]. The study by Farrell et al. [42] aimed to explore the host specificity of canopy nesting forms occurring in Queensland and New South Wales on two important host species, acacia and eucalyptus trees, by comparing the fecundity achieved on these host plants and testing the preference–performance hypothesis by reciprocal transplantion of egg masses and larval nests. The results showed that the nesting forms of *Ochrogaster lunifer* on acacia and eucalyptus trees differ somewhat with respect to the host plant groups.

The geographical variability of the cold tolerance of poikilothermic animals is one of the keys to understanding the mechanisms of the formation of their ranges under the influence of climate change or anthropogenic introductions. Meshcheryakova et al. [43] investigated the cold tolerance of the adult small tortoiseshell butterfly *Aglais urticae*, distributed from the Atlantic to the Pacific, which winters in a supercooled state in the north-east of Russia, compared to the previously investigated European species. Since the lower lethal temperature for this species is near −30 °C, the observed cold tolerance is not sufficient for *A. urticae* to overwinter in natural shelters above the snow cover over most of its range. Therefore, the settlement of *A. urticae* in regions with air temperatures below −30 °C is possible only if it hibernates under snow. This primitive behavioural adaptation probably does not require physiological changes and may not be exclusive to Lepidoptera.

The combined effects of local vegetation and landscape characteristics and the interactive effects of pollinators, pests, and their natural enemies on fruits and seeds set in an agroecosystem were studied by Shapira et al. [44]. The results showed that natural and semi-natural habitats can play a crucial role in mitigating the negative effects of aphid infestations on pollination services. Furthermore, they found that different groups of ecosystem service providers may respond differently to local landscape and habitat factors. These peculiar responses, observed in both pollinators and natural enemies, may be due to their unique biology and behaviour, different relationships with local or landscape food resources, or interactive effects. Chong et al. [45] studied the effects of different planting regimes and landscape compositions on the biodiversity and functional diversity of soil predators (spiders and carabids) in Jiangxi Province, China. The results indicated that the oilseed rape field increased the activity and diversity of carabid beetles, while the fallow land increased the activity and diversity of spiders, particularly the smaller and ballooning spiders. The winter oilseed rape field played a prominent role in maintaining the ground beetle diversity, while winter fallow was beneficial to spider diversity. Crespo et al. [46] identified the chemical composition and origin of the materials used to construct the outer shell of the secondary summer nests in *Vespa velutina nigrithorax*. They found that the nests are formed from cellulosic wood materials derived from surrounding softwood (gymnosperms) and hardwood (angiosperms) species, leaf debris, and even materials of agricultural origin. The beige stripes are formed almost exclusively from woody softwood cells, while the brown stripes consist mainly of hardwood cells, leaf tissue, and grasses. The elements C and O accounted for more than 99% of the chemical structure, and no significant mineral components were identified. This information is very important not only for understanding the biology of this hornet species in regions far from its area of origin but also for areas where this species could establish new colonies so that a possible future policy for biological control and control of invasive species can be established. This information could allow for populations to be fought without resorting to chemical control.

Characterisation and identification of a pest to determine its biodiversity can be the first step towards its control. El-Zoghby et al. [47] characterised the red palm weevil *Rhynchophorus ferrugineus*, one of the most damaging pests in palm cultivation, both morphologically and genetically, in five different geographical locations in Egypt. This study revealed no significant differences in body length and width between adults from the different sites, but different typologies of prothoracic spots indicated some diversity in the insect populations. Significant differences between the two sexes were found in the length of the antennal seta and forelegs, the length and width of the pronotum, and the length of the rostrum. Genetic variability was also found among the studied populations, and these differences could be due to the presence of different genotypes. The authors hypothesise that these findings could be important in developing a new strategy for tracking and controlling this invasive pest.

Insect-associated microbiota may also play an important role for their host, e.g., protection against pathogens, food supply, and survival in hostile environments. Cano-Calle et al. [48] studied the bacterial community of avocado thrips from northwestern Colombia to find isolates for potential biocontrol purposes. The authors reported that the diversity of the bacterial community in adult avocado thrips is low, with few bacterial components and the endosymbiont *Wolbachia* strongly predominant. They further suggested that this information is also relevant as a first step for biological and genetic control strategies in integrated pest management of thrips.

Living in a community with a social structure is one of the most important physical barriers against environmental elements and natural enemies, as the study by Uemura et al. [49] showed. Similar to various other insect shelters, the winter tent of *Thaumetopoea pityocampa* provides protection against environmental elements and natural enemies but also facilitates thermoregulation [50,51,52]. Social differentiation within the colony appears to be genetically determined. Wu et al. [53] found that in *Reticulitermes aculabialis*, structural analysis predicted that a specific protein, RaSsp1, binds and transports JH and that it plays an important role in the formation of soldier-specific traits, including head capsules and mandibles. They hypothesised that JH is secreted by the corpora allata and then bound to RaSsp1, which transports JH into the haemolymph and ultimately regulates soldier differentiation by activating its receptor Met and the transcription factor Kr-h1. Molecular principles also appear to underlie the high resistance of the red citrus mite, *Panonychus citri*, to spirodiclofen [54]. These results suggest that P450s and CCEs may be involved in the spirodiclofen resistance of *P. citri*. The transcriptome and RT-qPCR showed that CYP385C10 is the most upregulated gene in the spirodiclofen-resistant strain. RNAi data suggest that overexpression of CYP385C10 contributes to spirodiclofen resistance.

Rapid urbanisation is becoming an increasingly important driver of global change, leading to a decline in the abundance, diversity, and health of species and ecosystems. This is one of the biggest threats to arthropods, especially in densely populated geographic areas. Franzén et al. [55] studied the influence of local urbanisation on the composition of insect communities in two regions of Sweden to evaluate how changes in moth species richness, composition, diversity, and abundance are influenced by environmental conditions and species traits. The results suggested that factors such as habitat quality, availability of food resources, climatic conditions, and species interactions shape the assembly of moth communities, causing a decline in diversity. The 12-year ecological project, described in Deschamps-Cottin et al. [56], was based on the planting of host and nectariferous plants in Marseille at the Parc Urbain des Papillons. The results showed that it is possible to reverse the trend in biodiversity erosion. In fact, they found that planning a good management strategy increases species richness because vegetation structure strongly influences the butterfly communities.

The results of studies published in Animals indicate that the reclamation and restoration of habitats in highly anthropized areas are essential for the conservation of insect communities [57]. Luo et al. [58] analysed the effects of habitat fragmentation and vegetation composition on the ant community in the urban area of Nanchong, Sichuan Province, China. They found that ant species richness and ecological functional groups were influenced by vegetation community composition and seasonal variability. In addition, the study highlighted that urban greenery, forests, and small cultivated areas can be useful for mitigating the effects of anthropogenic pressure on the diversity of functional groups such as ants. Lin et al. [59] studied the distribution of butterfly species along an urbanisation gradient in the city of Fuzhou, Fujian Province, China. They found that the degree of urbanisation significantly affected species diversity, richness, and abundance. Furthermore, *Pieris rapae* and *Lampides boeticus* were identified as bioindicators of the impact of urbanisation on insect communities since they have low ecological requirements and a high tolerance to habitat fragmentation and temperature increase in urban areas. In contrast, ecologically more demanding species are mainly found in peri-urban areas.

Gas extraction is another anthropogenic activity that has disruptive effects on the insect community. Curran et al. [60] observed the abundance and diversity of several families of Hymenoptera, Hemiptera, Coleoptera, Lepidoptera, Orthoptera, and Diptera in wells at the Jonah Infill natural gas field, Sublette County, WY, USA. They found that the configuring of patches of vegetation with native flowering plants around extraction sites has the potential to mitigate the harmful effects on insect communities that are responsible for providing essential ecosystem services.

Xu et al. [61] aimed to determine the population differentiation of *Spirobolus bungii* in eastern and northern China and reveal the genetic structure and diversity of millipedes and the factors influencing them. The results showed that gene flow between populations of *S. bungii* increased the consistency of genetic structure. However, large geographical barriers, such as between the Yangtze River and the Yellow River, significantly blocked gene flow between *S. bungii* populations, resulting in a high degree of genetic differentiation. Zang et al. [62] described most life stages of a new *Limnephilus* species from Qinghai Province, China, by molecular analysis and provided ecological information on this new species. In this study, based on male genitalia and following Schmid’s system, the authors reviewed all *Limnephilus* species from mainland China at the species group level, highlighting their morphological characteristics. It is known that there is a strong relationship between morphological characteristics and function [63]. The study by Whalen et al. [64] tested the hypothesis that individuals maintain symmetry in the structures that are most important for maximising fitness based on their specific life strategy. Direct selection on genetically determined polymorphisms is probably the strongest form–function relationship; however, polyphenic species also exhibit environmental interactions. In such cases, alleles at polyphenic loci are an essential component of form determination, but so are a variety of environmental factors such as nutrition, population density, tactile cues, infection, and injury [65,66,67].

The exponential growth of the human population has led to a corresponding increase in crop production, which in turn has necessitated the development of complex pest control strategies [68]. The role of the olfactory system in insects for host plant selection is widely recognised, with the antenna being the most important organ of *Spodoptera frugiperda* for the perception of odours. The study by Wang et al. [69] comprehensively documented antennal sensilla in the larvae of the invasive pest *Spodoptera f.*, which include olfactory pores, sensilla pegs, and five types of sensilla. They also identified olfactory pores and described 12 types of different sensilla on the antennae of adult insects. Although the physiological function and the role of individual sensilla in host localisation are not yet fully understood, the authors suggest that this information could also be crucial in this case for the development of new control strategies to effectively manage this invasive pest. The tobacco cutworm, *Spodoptera litura*, is a common phytophagous pest of crops worldwide. In their study, Hu et al. [70] analysed seven microsatellite loci as molecular markers to investigate the genetic diversity and population differentiation within 24 geographic populations of this species. Their aim was to develop a strategy to control the spread of this pest in localised areas. The results indicated that high levels of gene exchange and low genetic differentiation in maintaining local population diversity may contribute to population outbreaks and insecticide resistance. In the study conducted by Zayed et al. [71], the larval stages of *Spodoptera littoralis* were exposed to a solution of microorganisms, including lactic acid bacteria, photosynthetic bacteria, yeasts, actinomycetes, and fungi used to promote plant growth. Antifeedant activity, food uptake index, efficiency of digested food, and relative growth rate were tested as markers for exposure effects. All tested concentrations affected the physiological processes of the larvae and influenced their life cycle and development. The results showed that the tested formulation has the potential to be used as a bioinsecticide to replace conventional chemical control methods in integrated pest management.

Specialisation on host plants may be a precursor of local adaptation and a possible factor of speciation. Many species have a plasticity in their diet that allows them to adapt to new environments [72]. The insect *Formica paralugubris* utilises two traits to adapt to new environments: the plasticity of its diet and the ability to use different nest mound materials depending on the habitat in which it resides [73,74]. Frizzi et al. [73], studying the ability of this specie to adapt to the Apennine environment, found that the difference in facial colouration between introduced populations and their populations of origin may be hereditary, given the clear similarity between the native Alpine sites and those introduced. Interestingly, this phenotypic trait appears to be quite conservative, as it has not changed in the ants of introduced populations in the six decades since their introduction, despite the different habitats in which they live. This suggests a genetic background in the expression of this trait that requires long-lasting adaptive pressure to be modified at the population level. The study by Solari et al. [75] shed light on the morphological and functional organisation of the stereotyped rhythmic motor pattern known as “calling behaviour” in *Lymantria dispar*. In particular, they proposed that the release of sex pheromones in the female spongy moth occurs through the extension and retraction of the ovipositor, which is controlled by a coordinated motor programme maintained mainly by the activity of a few motor units under the control of TAG nerves N4 and N5, previously found to be octopaminergic [76].

Although anthropogenic global change is causing a sustained decline in biodiversity on the global scale, the efforts of researchers involved in monitoring activities often lead to the identification and morphological description of new species. A new species, *Bryaxis aetnensis* (Coleoptera: Staphylinidae), found in volcanic caves of Mount Etna (Italy, Sicily), has been described, and diagnostic characters for males and females have been provided [77]. The discovery of this species highlights the importance of protected areas for habitat conservation. Klimov et al. [78] described five new species of *Thyreophagus* (Acari: Acaridae) and provided a diagnostic key for females, males, and heteromorphic deutonymphs of the genus, leading to new insights into reproduction in mites. Morphological analyses showed that four of the described asexual species retain functional copulatory and sperm storage systems, suggesting that these lineages have a relatively short evolutionary lifespan. Defilippo et al. [79] studied the species composition and density of sand flies in Lombardy (Northern Italy) with the aim of obtaining data on the distribution of leishmaniasis vectors through systematic entomological surveillance. They found the presence of several species collected in low-altitude areas that could contribute to the spread of pathogens, as documented by the occurrence of epidemic outbreaks of leishmaniasis and other sand fly-borne diseases in areas previously considered non-endemic [80]. Silva et al. [81] investigated the sand fly species in João Pessoa, in forested and built-up areas, comparing the diversity and abundance of these areas and their relationship with environmental conditions. They found that there are differences in the richness and diversity of phlebotomine species in the built-up areas compared to the forested areas. In addition, the study showed that the conservation of forested areas, even in urban fragments, favours the diversity of the sand fly fauna.

Two articles published on this topic address the taxonomy and phylogenetic classification of species. In particular, given the interest in the gooseneck barnacles *Conchoderma* due to their ability to grow on a wide variety of marine organisms [82], Chan and Chen [83] studied the morphology and sequence divergence in the COI gene of *C. hunteri* and *C. virgatum* to determine their still controversial taxonomic status. Overall, both morphological and molecular evidence confirmed that they can be considered as two distinct species. In the second study, Ye et al. [84] sequenced the complete mitochondrial genomes of two *Lycosa* wandering spiders, *L. shansia* and *L. singoriensis*, that live mainly on forest floors and are the main predators of agricultural pests [85], to investigate the phylogenetic position and mitogenomic composition and evolution of these species. The authors concluded that the mitogenomic structures of *L. shansia* and *L. singoriensis* are the same as those of the other Lycosoidea species and showed the position of the two Lycosidae species in the phylogenetics.

X-ray computed microtomography has become an important imaging technique used in morphological studies as well as in taxonomic, phylogenetic, and ecological contexts [86,87,88,89,90,91,92]. It is suitable for faster data acquisition of three-dimensional (3D) imaging data compared to classical histological analyses. This method was used to analyse the morphology of compound eyes in four coleopteran species—*Clinidium canaliculatum* (Rhysodidae), *Tenebrio molitor* and *Tribolium castaneum* (Tenebrionidae), and *Pterostichus melas italicus* (Carabidae)—which inhabit different habitats and have different ecological functions [93]. Virtual sections and 3D renderings of the head enabled non-invasive measurements of eye morphological parameters such as interocular distance, facet density, corneal thickness, and number of ommatidia to understand how different lifestyles and eye and brain morphology have co-evolved under the selective pressure of biotic (food, predators) and abiotic (light) factors.

Finally, the reviews by Gonzalez-Ponce et al. [94] focused on scorpions, which, despite their life-threatening venom, have been adopted as part of the landscape and daily life by the citizens of the city of Durango in Mexico, incorporating them into their folklore and economic resources and learning how to benefit from their abundance. The diverse uses of scorpions include basic scientific, clinical, and biological applications and beyond. Their venom is lethal, but under certain conditions it could be used for therapeutic purposes and/or in many biotechnological applications. Scorpions themselves represent a protein-rich food source or a model to study exoskeleton fluorescence, survival skills, and other unexplored traits. Therefore, these authors suggested that many bio-based economic resources can be positively impacted by expanding our knowledge about scorpions. The research progress on the “3-tropism” of ants (consisting of phototaxis, chromotaxis, and chemotaxis) was discussed and systematically summarised in the review by Dong et al. [95] to guide research and future production of environmentally friendly, green, and harmless methods for the prevention and control of ants in households and fields. In the review of global rice stem borer species [96], it was reported that among lepidopteran stem borer species, assemblages are structured by the presence of a single, highly dominant species that feeds mainly on rice for which the growing system (including water availability and temperatures) is optimal and the growth period is long enough to allow complete larval development; assemblages may contain one or more oligophagous or polyphagous secondary species that may dominate the resource in terms of age, anatomy (e.g., stem thickness, tiller number), or proximity to key habitats and crops (e.g., *S. inferens* in wheat-rice systems). Finally, populations may occasionally contain oligophagous or polyphagous species that are of little economic importance for rice production and probably originate from native grasslands or other habitats.

## Data Availability

Not applicable.
